# The impact of desmoid tumors: insights from the Desmoid Tumor Research Foundation Natural History Study, 2017–2023

**DOI:** 10.1186/s13023-025-04021-7

**Published:** 2025-10-14

**Authors:** Amanda Lucas, Shengfan Zhou, Lynne Hernandez, Timothy Bell, Ana B. Oton, Kelly Mercier

**Affiliations:** 1 Desmoid Tumor Research Foundation, Woodcliff Lake, NJ USA; 2 SpringWorks Therapeutics Inc., Stamford, CT USA; 3 Department of Orthopedic Surgery, Duke University Medical Center, Durham, NC USA

**Keywords:** Aggressive fibromatosis, Desmoid tumor, DTRF, Natural history study, Patient journey, Patient-reported outcomes, Quality of life, Rare tumor, Real-world data, Sarcoma

## Abstract

**Background:**

Desmoid tumors (DT) are rare, locally aggressive soft tissue tumors that pose considerable diagnostic and treatment challenges, and may negatively impact patients’ overall quality of life (QoL). This analysis examines three aspects of the real-world patient experience with DT: (1) demographics and disease characteristics, (2) the journey from initial signs and symptoms to definitive DT diagnosis and treatment, and (3) QoL. The ongoing Desmoid Tumor Research Foundation (DTRF) Patient Registry and Natural History Study (NHS) examines patient-reported survey data to characterize the real-world nature and impact of DT, including clinical history, prior and current treatment, and QoL. The GOunder/DTRF Desmoid Symptom/Impact Scale (GODDESS^©^) patient-reported outcomes tool was used to assess DT-specific QoL, such as pain, fatigue, physical functioning, sleep, and emotional impact.

**Methods:**

This analysis was performed on data collected by the DTRF NHS between September 2017 and August 2023 using the most recent survey submitted by participants, including clinical characteristics, treatment experience, and patient-reported outcomes (PROs) data assessed by the GODDESS tool. Where applicable, continuous variables were assessed using descriptive statistics. Between-group proportions were compared using Pearson’s chi-square test or Fisher’s exact test. Analysis of variance was performed to assess the differences in mean time to diagnosis and mean scores of GODDESS Desmoid Tumor Symptom Scale (DTSS) and Desmoid Tumor Impact Scale (DTIS) domains and items across the subgroups of age, current tumor status, and tumor location where appropriate.

**Results:**

Surveys were completed by 399 participants between September 2017 and August 2023. The median age of participants was 38 years; most were female (72%, 288/399), White (84%, 334/399), or resided in North America (86%, 345/399). Signs and symptoms reported at diagnosis included an unexplained bump (60%, 238/399), pain (57%, 226/399), and fatigue (14%, 54/399). A total of 162 participants (41%, 162/399) reported a prior misdiagnosis. Surgical procedures were the most common first-line therapy in those reporting multiple treatments for DT (59%, 74/126), followed by chemotherapy (24%, 30/126). Among 126 participants who reported a prior misdiagnosis and history of surgery for DT, 46% (58/126) reported having received surgery before a definitive DT diagnosis. For those undergoing surgery (any line) post-diagnosis, tumor recurrence was common (63%, 103/163). Participants with the presence of DT at survey completion reported significantly worse scores for pain, extra-abdominal symptoms, fatigue, physical functioning, and sleep in comparison to those without the current presence of a DT (*P* < 0.05).

**Conclusions:**

Patients with DT can experience a high disease burden. Misdiagnosis can lead to unnecessary surgery, potentially delaying an appropriate treatment for DT, and tumor regrowth that can contribute to impaired QoL after surgery. The analysis of NHS data presented in this manuscript highlights real-world experiences of patients with DT and underscores the need for patient-centric treatment strategies and outcomes.

## Background

Desmoid tumors (DT) are rare and intermediate (locally aggressive) soft tissue tumors that occur in the connective tissue [1–3]. DT are typically large and bulky masses that can be 5–10 cm in longest dimension, although some may be larger [4, 5]. The disease course is unpredictable and varies by tumor location, which poses significant diagnostic and treatment-related challenges [4, 6, 7]. Although non-metastatic, DT can be multifocal and locally infiltrative, leading to substantial morbidity and negatively impacting patients’ functional level and overall quality of life (QoL) [1, 4, 6]. As a result of the life-changing and lifelong nature of the condition, patients with DT can also face impactful psychological and social burdens [6].

Patients possess unique knowledge and expertise in characterizing their disease or condition. Accordingly, patient-reported data about DT can help deepen the understanding of health status and disease state [6]. This may enable clinicians to individualize patient care and ultimately help transform the healthcare experience for patients with DT [6]. Therefore, greater insight is needed into patient experiences with the medical assessments and procedures performed before DT diagnosis, the time to achieve a confirmed diagnosis, and subsequent experiences with treatment, clinical outcomes, and QoL. Due to the limited patient-reported data for DT, ongoing data collection is essential to better understand the natural history and real-world effects of DT treatments [6]. The Desmoid Tumor Research Foundation (DTRF) Patient Registry and Natural History Study (NHS) is the largest DT-specific international natural history study to date [8]. Beyond the DTRF NHS, no other comprehensive dataset exists that uses internationally-sourced patient-reported survey data to characterize the real-world nature and impact of DT [8]. The objective of this study was to gain further insight into three key aspects of the real-world disease burden and patient journey for those with DT using DTRF NHS data: (1) demographics and disease characteristics, (2) the journey from initial signs and symptoms to definitive DT diagnosis and treatment, and (3) QoL.

## Methods

The DTRF NHS is an ongoing, prospective, longitudinal study of patients worldwide who have a self-reported diagnosis of DT and comprises several surveys developed by the National Organization for Rare Disorders (NORD) and DTRF NHS investigators [8]. These DT-specific surveys gather patient-reported information regarding clinical characteristics (tumor characteristics, initial signs and symptoms, and diagnosis), prior and current treatments (including sequencing and outcomes), disease monitoring, and patient-reported QoL [8], in addition to demographic data. Surveys were completed online by adults with DT or by the legally authorized representatives of participants aged < 18 years. All survey questions were optional. Participants were made aware of the surveys via DTRF representatives, clinicians, or by independently visiting the DTRF website to complete the surveys. Ethical oversight and approval for the DTRF NHS was obtained from the North Star Review Board (NB100030). The study was conducted in accordance with guidance from the International Council for Harmonisation of Technical Requirements for Pharmaceuticals for Human Use (ICH), Good Clinical Practice (GCP), and the United States Code of Federal Regulations (CFR). The study was also performed in accordance with the Helsinki Declaration of 1964 and its later amendments. Informed consent was obtained electronically as part of the DTRF NHS registration process, prior to collecting any study-related data.

QoL was measured using the GOunder/DTRF Desmoid Symptom/Impact Scale (GODDESS^©^), a validated DT-specific PRO tool. The GODDESS Desmoid Tumor Symptom Scale (DTSS) measures DT-specific symptom severity (over the previous 24 h) and the Desmoid Tumor Impact Scale (DTIS) measures sleep, physical functioning, and the emotional impact on the lives of participants (over the previous 7 days) [9]. GODDESS DTSS and DTIS comprise several domains, with each domain constituted by various items (i.e., questions). An 11-point numeric rating scale (0 to 10) was used to assess the GODDESS DTSS domains and the GODDESS DTIS Emotional Impact domain, where 0 represents “none” and 10 represents “as bad as you can imagine” [9]. A 5-point Likert scale (0 to 4) was used to assess the GODDESS DTIS Physical Functioning and Sleep domains, where 0 means “none of the time” and 4 means “all the time” [9]. For all domains, higher scores indicate worse symptoms or more severe impact [9].

Domains assessed by the GODDESS DTSS include Pain (items include Worst Pain, Dull Pain, and Shooting Pain), Extra-Abdominal Symptoms (items include Swelling, Muscle Weakness, and Difficulty Moving Body Areas Near the Tumor), and Intra-Abdominal Symptoms (items include Nausea, Abdominal Pain, and Feeling of Fullness After Beginning to Eat). Fatigue was assessed as a single item [9]. A Total Symptom Score was also calculated based on DTSS items using the following formula [9]:

$$\begin{gathered} \frac{{\left( {\frac{{Pain + Dull\:Pain + Shooting\:Pain}}{3}} \right) + Swelling} + {Muscle\:Weakness + Difficulty\:Moving + Fatigue}}{5} \hfill \\ \end{gathered} $$

Domains assessed by the GODDESS DTIS include Physical Functioning (items include Difficulty Moving Body Areas Near the Tumor, Difficulty Reaching Up, Difficulty Doing Moderate Activities, Difficulty Doing Vigorous Activities, and Accomplishing Less Daily Activity), Sleep (items include Difficulty Getting Comfortable in Bed, Trouble Falling Asleep, and Trouble Staying Asleep), and Emotional Impact (items include Fear of Future Diagnostic Tests, Fear of Desmoid Tumor Growth/Recurrence, Hopelessness, Anger, Anxiety, and Frustration) [9].

The analysis in this study was based on data collected between September 2017 and August 2023. Where applicable, continuous variables were assessed using descriptive statistics; proportions were compared between groups using Pearson’s chi-square test or Fisher’s exact test. Analysis of variance was used to assess differences in mean time to diagnosis and the mean scores of DTSS and DTIS domain and items across the subgroups of age, current tumor status, and tumor location. All data analyses were generated using SAS version 9.4 (Cary, NC, USA). Note that *N*-values vary among analyses due to differing data completeness across survey questions.

The sample size for each subgroup was determined by data availability, and a sample size of 10 or more participants was required to be considered for the analyses. Not all participants reported all data, and missing data were excluded from subgroup analyses. Participants could report more than one option for signs or symptoms, tumor location, incorrect diagnosed conditions, and treatment so that the categories were not mutually exclusive; therefore, cumulative percentages do not total 100%.

## Results

**Fig. 1:**
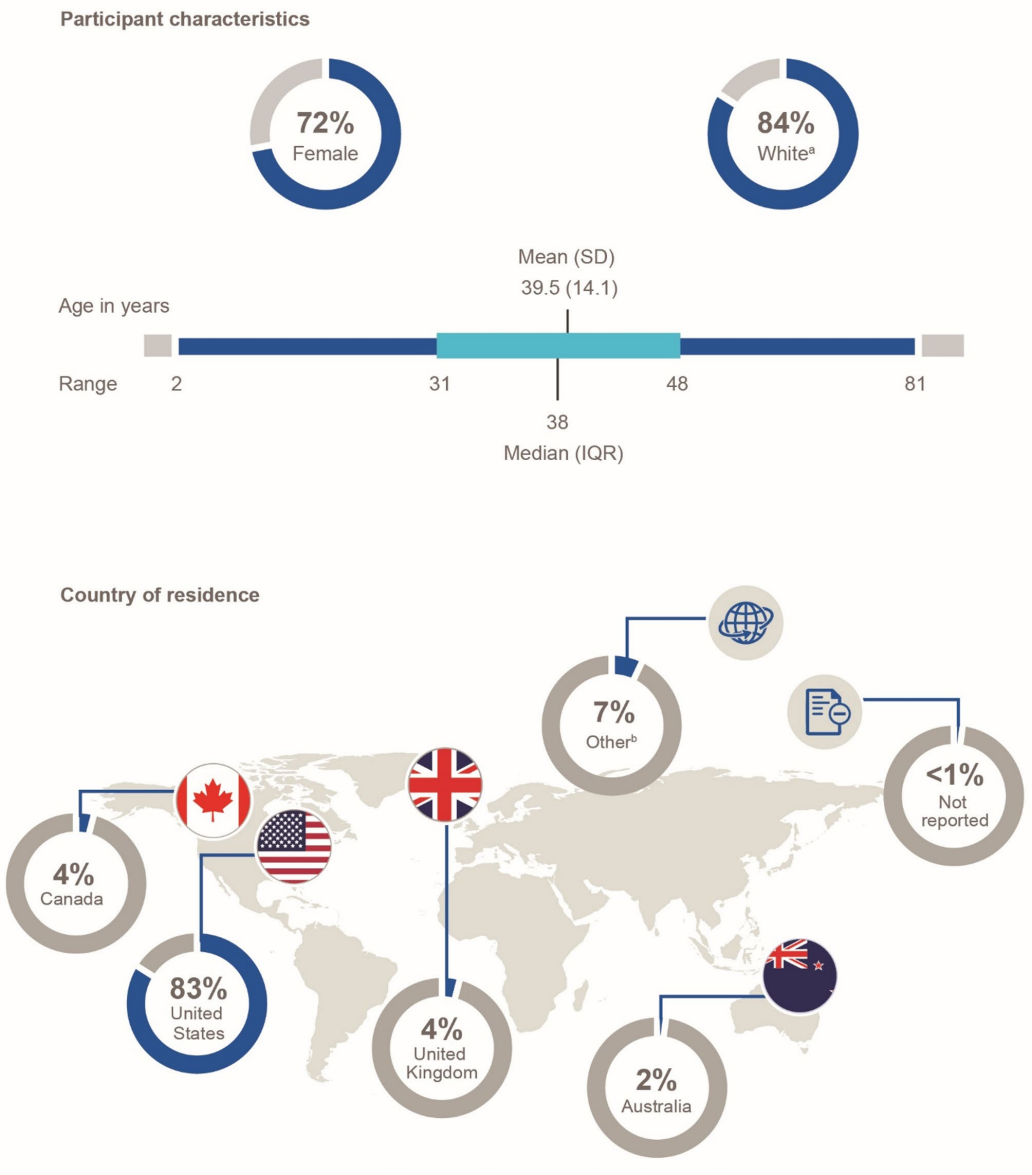
Participant demographics (*N* = 399). ^a^Race also includes American Indian or Alaska Native (3), Asian (7), Black or African American (13), Missing (30), Native Hawaiian or Pacific Islander (1), Other (9), Refused (1), and Unknown (1). ^b^Twenty-four additional countries with fewer than 10 participants comprised Argentina (1), Belgium (1), Botswana (1), Brazil (2), the Cayman Islands (1), Denmark (1), Estonia (1), France (2), Germany (1), Guatemala (1), Hong Kong (1), India (1), Ireland (2), Italy (2), Japan (1), Jersey (1), Jordan (1), the Netherlands (1), Norway (1), the Philippines (1), Poland (1), Romania (1), Sweden (1), and the United Arab Emirates (1). IQR, interquartile range; SD, standard deviation

A total of 399 participants completed the DTRF NHS surveys during the specified study window. At the time of data cutoff, the median age of participants at survey completion was 38 years (93% adults); most were female (72%), White (84%), or resided in North America (86%; Fig. 1).

### Participant journey to DT diagnosis

**Fig. 2:**
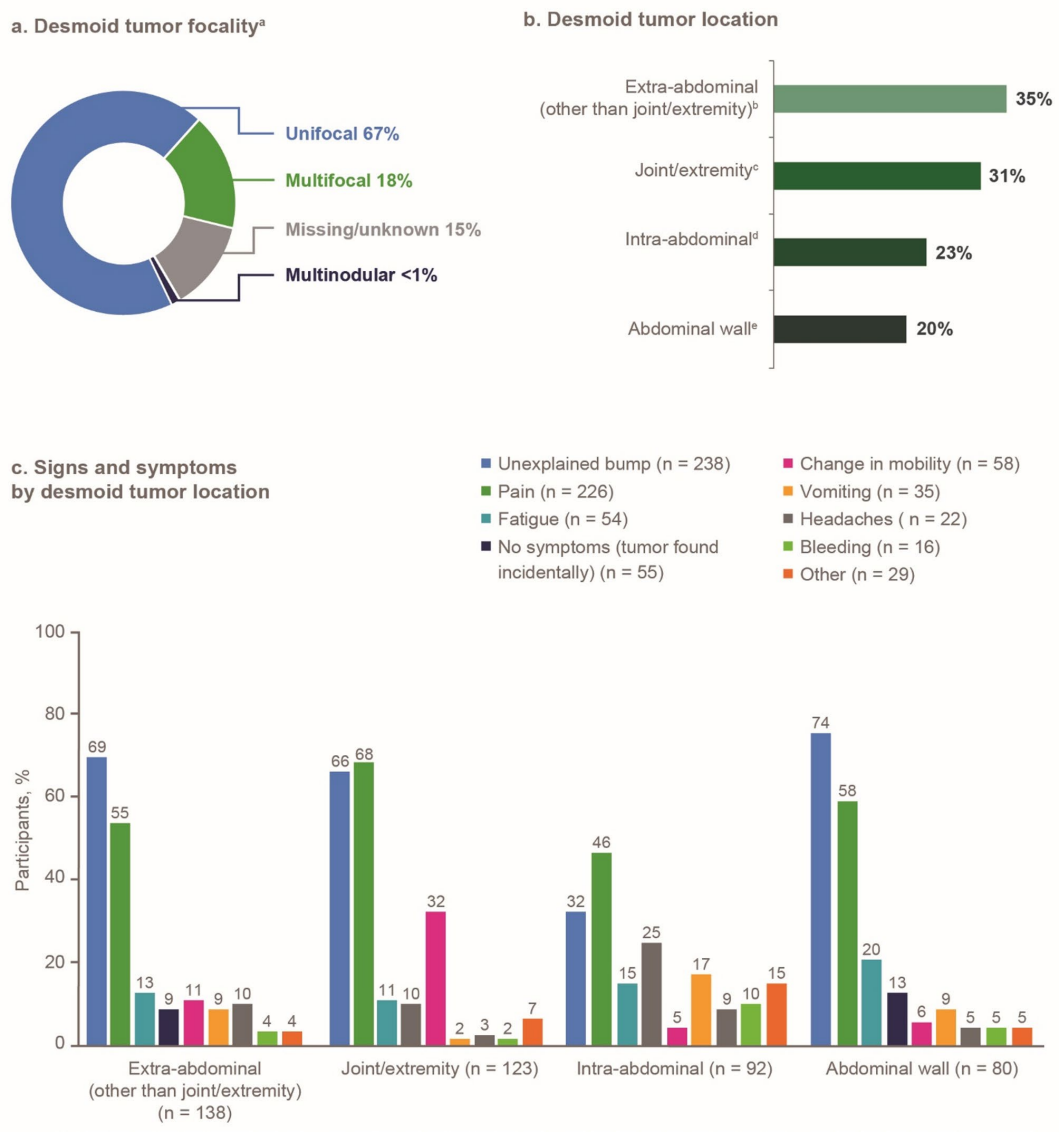
Desmoid tumor characteristics and signs and symptoms at diagnosis (*N* = 399). Participants could report multiple symptoms and tumor locations at diagnosis; therefore, percentages do not total 100%. ^a^Percentages have been rounded and therefore may not total 100%. ^b^Extra-abdominal refers to the head/neck, chest wall, and other locations not included in the remaining categories. ^c^Joint/extremity refers to the hips, knees, shoulders, arms, hands, feet, and legs. ^d^Intra-abdominal refers to locations deep in the abdomen and involving the bowels, kidney, and/or pelvis. ^e^Abdominal wall refers to a superficial location on the stomach muscle

At the time of diagnosis, the median tumor size was 5.7 cm (longest DT dimension, *n* = 201), and 67% (267/399) of tumors were unifocal (one or more tumors at a single anatomic location) compared with 18% (72/399) reporting multifocal tumors (15% unknown/missing; Fig. 2a). The most common tumor locations were extra-abdominal (35%, 138/399; head, neck, chest wall, and other than joint/extremity), and joint/extremity (31%, 123/399; hips, knees, shoulders, arms, hands, feet, and legs; Fig. 2b). Unexplained bump (60%, 238/399) and pain (57%, 226/399) were the most common sign or symptom of DT, regardless of tumor locations (Fig. 2c).

The median age at the first sign or symptom of DT was 32 years, and the average time from the first sign or symptom to confirmed DT diagnosis was 4 years (*n* = 292; median = 1 year). Most DT diagnoses (70%) were received within 1 year after the appearance of the first sign or symptom, compared with 1–2 years (12%), 2–5 years (8%), and > 5 years (11%). The time from the first sign or symptom to confirmed DT diagnosis differed significantly by age and tumor location. Participants aged ≤ 30 years at their first sign or symptom experienced a significantly longer mean time to diagnosis (7 years) compared with 1 year for participants > 30 years, (*P* ≤ 0.0001), as well as those with intra-abdominal tumors (8 years) compared with 3 and 4 years, respectively, for joint/extremity and abdominal wall and extra-abdominal tumors (*P* = 0.024).

Participants frequently (41%, 162/399) reported receiving a misdiagnosis, regardless of tumor size, location, or focality (Table 1). The most reported differential diagnoses (70%, 113/162) were benign tumors or other conditions, including muscle injury, lipoma, colon polyps, postoperative scars, early-onset arthritis, hernia, and Baker’s cyst. Participants also reported misdiagnoses of sarcoma (17%, 28/162; including gastrointestinal stromal tumor [GIST]) and other cancers (17%, 28/162; including lymphoma, vascular tumor, and breast cancer).

**Table 1:** Participant clinical characteristics

Clinical characteristics^a^	Overall *N*	Incorrectly diagnosed *n* (%)	Correctly diagnosed *n* (%)
All participants^b^	399	162 (41)	232 (58)
Sex^c^	389	160 (41)	229 (59)
Male	106	32 (30)	74 (70)
Female	283	128 (45)	155 (55)
Age at first symptom	394	162 (41)	232 (59)
≤ 30 years	197	76 (39)	121 (61)
> 30 years	197	86 (44)	111 (56)
Longest desmoid tumor dimension	197	87 (44)	110 (56)
< 10 cm	144	59 (41)	85 (59)
≥ 10 cm	53	28 (53)	25 (47)
Desmoid tumor focality	335	132 (39)	203 (61)
Unifocal	265	105 (40)	160 (60)
Multifocal	70	27 (39)	43 (61)
Desmoid tumor location^d^	383	160 (42)	223 (58)
Extra-abdominal (other than joint/extremity)	135	49 (36)	86 (64)
Joint/extremity	123	61 (50)	62 (50)
Intra-abdominal	91	40 (44)	51 (56)
Abdominal wall	79	30 (38)	49 (62)
Surgery before diagnosis	310	126 (41)	184 (59)
Yes	127	58 (46)	69 (54)
No	183	68 (37)	115 (63)

^a^*N*-values vary for these analyses due to missing data for some participants. ^b^5 participants were missing misdiagnosis data. ^c^The percentage of participants misdiagnosed when examined by sex (i.e., male vs. female) is statistically significant (*P* = 0.007). Two participants identified as transsexual. ^d^Some participants also report more than one option; therefore, percentages do not total 100%

### Participant journey from DT diagnosis to treatment

**Fig. 3:**
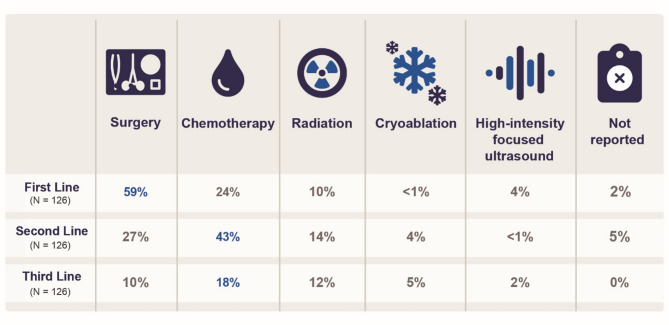
Desmoid tumor treatment sequencing for participants who reported multiple treatments (*N* = 126)

Following a confirmed diagnosis of DT, a total of 383 participants completed the treatment survey, with 136 (36%) reporting active surveillance before treatment initiation. Approximately half (53%, 202/383) received DT treatment, including surgery (43%, 163/383), which was the most common first-line therapy among participants who received multiple treatments (33%, 126/383; Fig. 3). Among 126 participants who reported a prior misdiagnosis and history of surgery for DT, 46% (58/126) reported having received surgery before a definitive DT diagnosis. Most participants (76%, 281/369) received systemic therapy (any line), including nonsteroidal anti-inflammatory drugs (NSAIDs; 40%, 112/281), tyrosine kinase inhibitors (32%, 91/281), chemotherapy (31%, 87/281), and hormone antagonists (17%, 47/281; Table 2).

**Fig. 4:**
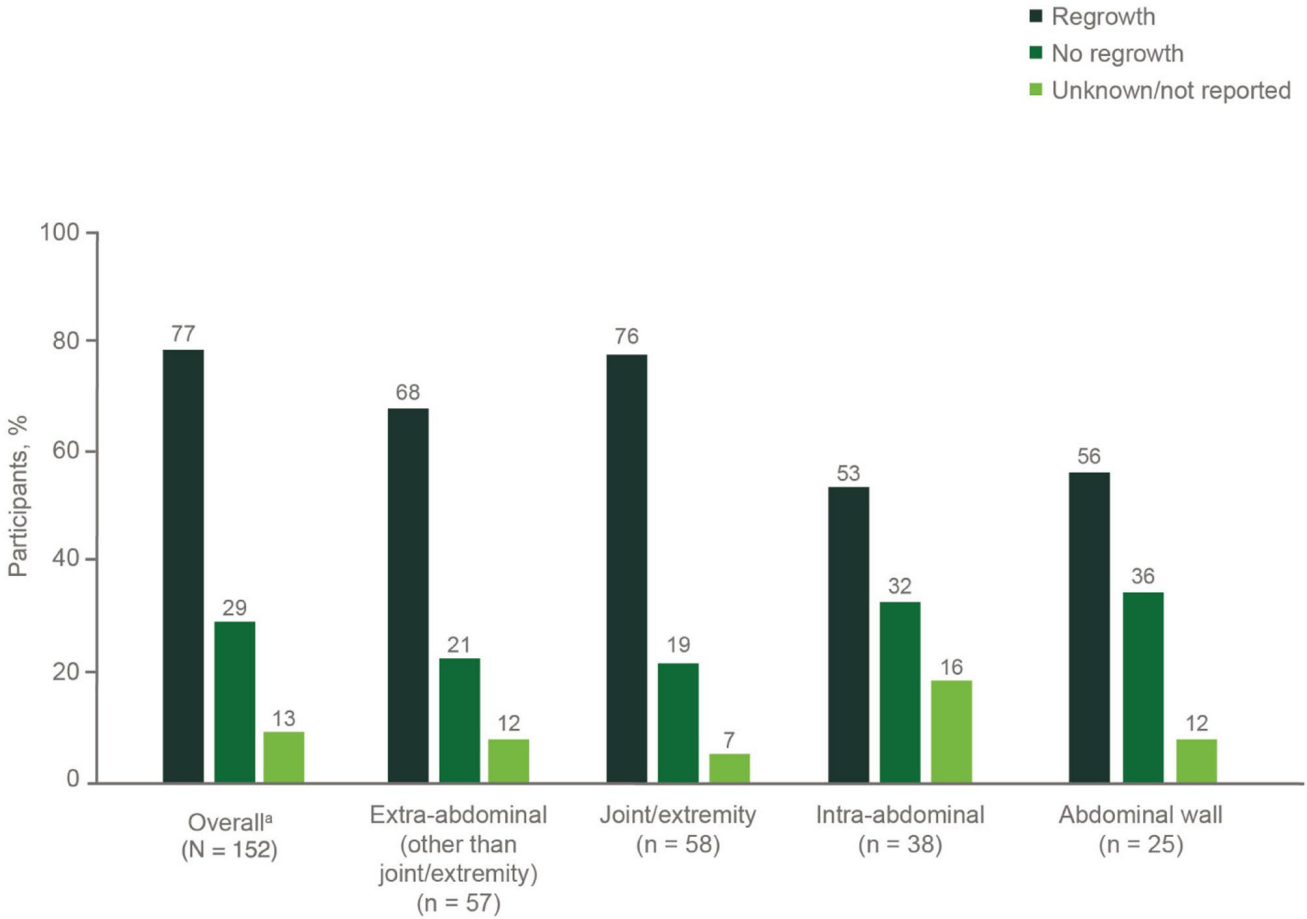
Surgical outcomes by tumor location. ^a^Some participants reported more than one tumor location; therefore, percentages exceed 100%

Continued tumor regrowth or recurrence following any DT treatment was reported for 46% (92/202) of participants, with a higher rate post-surgery (63%, 103/163), regardless of tumor location (Fig. 4). Amputation was reported for 7% (12/163) of participants who underwent surgery, with most (83%, 10/12) experiencing tumor regrowth or recurrence post-amputation.

**Table 2:** Desmoid tumor systemic treatments, any line (*N* = 281)

Systemic treatments	*n* (%)
Chemotherapeutics	87 (31)
Methotrexate	32 (37)
Vinblastine	26 (30)
Doxorubicin	25 (29)
Liposomal doxorubicin	25 (29)
Dacarbazine	7 (8)
Vinorelbine	6 (7)
Hydroxyurea	2 (2)
Ifosfamide	2 (2)
NSAIDS (with sulindac and celecoxib)	112 (40)
NSAIDS (not otherwise specified)	51 (46)
Sulindac	46 (41)
Celecoxib	23 (21)
Tyrosine kinase inhibitors	91 (32)
Sorafenib	64 (70)
Imatinib	26 (29)
Pazopanib	9 (10)
Sunitinib	1 (1)
Hormone antagonists	47 (17)
Antihormonal agent (e.g., tamoxifen)	41 (87)
Toremifene	4 (9)
Anastrozole	2 (4)
Gamma-secretase inhibitor	3 (1)
Other (e.g., rituximab)	57 (20)

Percentages in main category rows are based on *N* = 281. Percentages in sub-category rows are based on *n*-values indicated in the preceding main category row. Note that nirogacestat was not approved for use at the time of survey completion [18], and hormone antagonists are no longer recommended by Desmoid Tumor Working Group Consenus Guidelines [13]

NSAIDs, nonsteroidal anti-inflammatory drugs

Most participants (70%, 240/341) reported the current presence of DT at the time of survey completion. Regardless of the current presence of DT, the use of medications for symptom management was similar (Table 3). As expected, a higher proportion of participants (77%, 181/235) with a current DT were monitored (regular clinic visits and radiologic assessment, including computed tomography and magnetic resonance imaging) at least every 6 months relative to those without (54%, 49/91). Participants with familial adenomatous polyposis (FAP) and those with *CTNNB1* mutations had a similar frequency of monitoring (*P* = 0.694).

**Table 3:** Medications for desmoid tumor symptom management

Medications	Overall (*N* = 322)	Current presence of a desmoid tumor	
		Yes (*n* = 225)	No (*n* = 97)
	*n* (%)	*n* (%)	*n* (%)
NSAIDs^a^	151 (47)	103 (46)	48 (49)
Antidepressants^b^	60 (19)	41 (18)	19 (20)
Anticonvulsants^c^	36 (11)	26 (12)	10 (10)
Muscle relaxants^d^	39 (12)	28 (12)	11 (11)
Opioids^e^	61 (19)	45 (20)	16 (16)
Other	33 (10)	26 (12)	7 (7)
None	104 (32)	69 (31)	35 (36)

^a^Acetylsalicylic acid, celecoxib, ibuprofen, indomethacin, naproxen, oxaprozin, and nabumetone. ^b^Sertraline, fluoxetine, citalopram, escitalopram, paroxetine, fluvoxamine, and trazodone. ^c^Carbamazepine, diazepam, ethosuximide, and gabapentin. ^d^Baclofen, chlorzoxazone, carisoprodol, cyclobenzaprine, dantrolene, diazepam, metaxalone, methocarbamol, and tizanidine. ^e^Codeine, fentanyl, hydrocodone, meperidine, and methadone

NSAIDs, nonsteroidal anti-inflammatory drugs

### The impact of DT on QoL

**Fig. 5:**
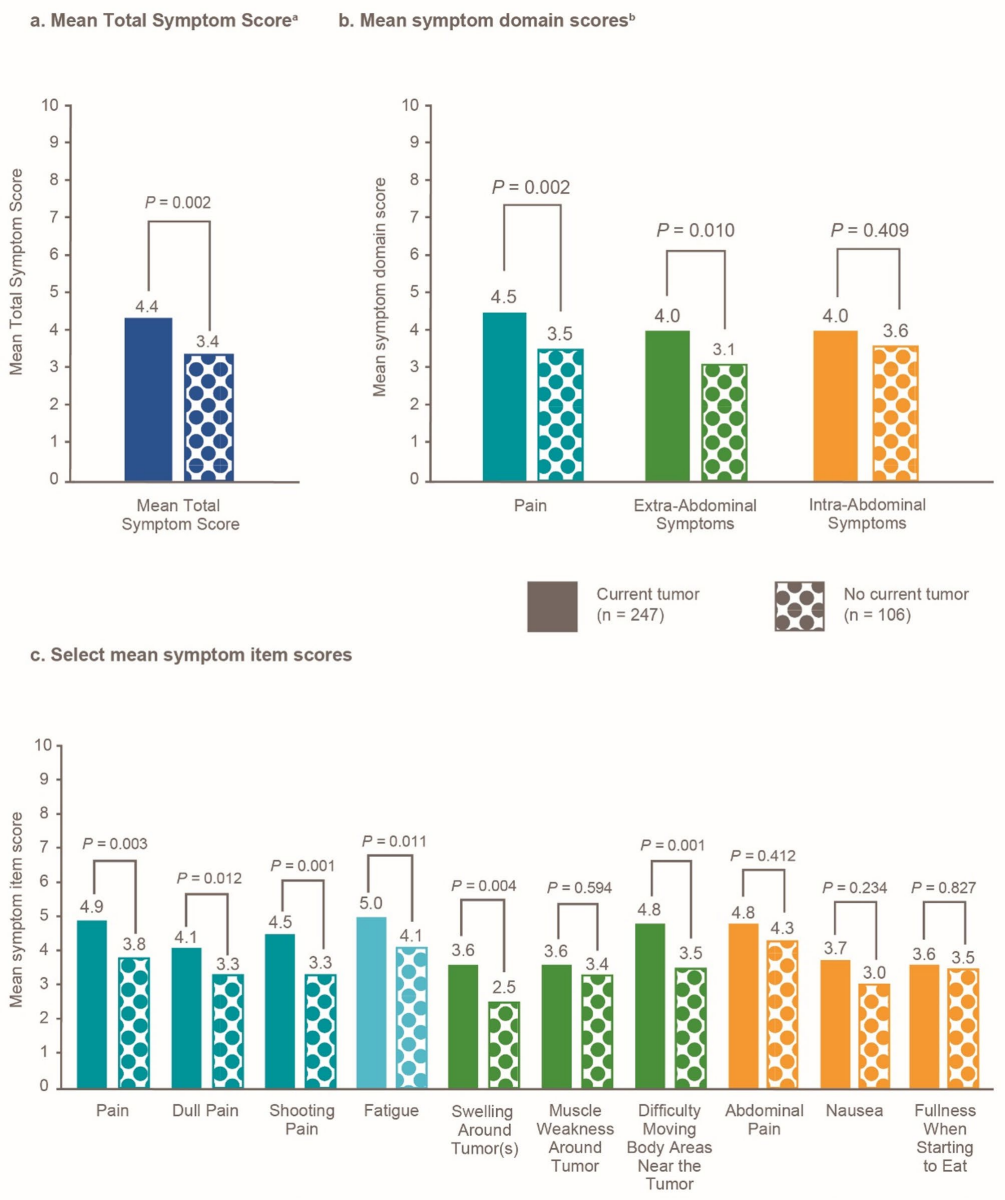
GODDESS DTSS symptom scores by tumor status. ^a^The overall mean Total Symptom Score for all participants was 4.1. ^b^The overall mean scores for all participants were: Pain, 4.2; Extra-Abdominal Symptoms, 3.8; and Intra-Abdominal Symptoms, 3.9. Colors are used to link the domains with their constituent items. DTIS, Desmoid Tumor Impact Scale; GODDESS, GOunder/DTRF Desmoid Symptom/Impact Scale

**Fig. 6:**
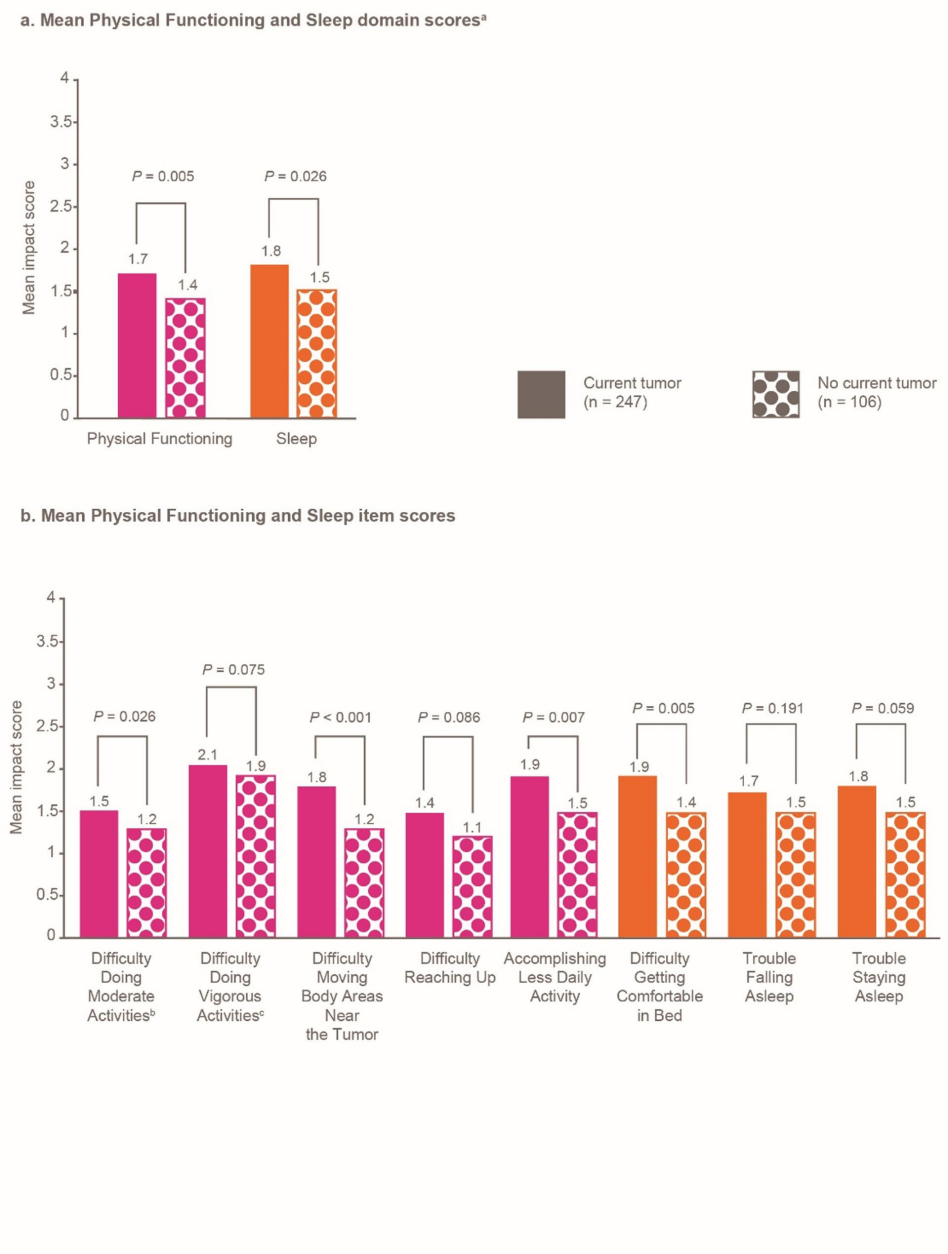
GODDESS DTIS impact scores by tumor status. ^a^The overall mean scores for all participants were: Physical Functioning, 1.6; and Sleep, 1.7. ^b^Moderate activities include pushing a vacuum cleaner, playing with children, and taking a long walk. ^c^Vigorous activities include running, lifting heavy objects, and participating in strenuous sports. Colors are used to link the domains with their constituent items. DTIS, Desmoid Tumor Impact Scale; GODDESS, GOunder/DTRF Desmoid Symptom/Impact Scale

Participants with the current presence of DT reported significantly worse mean scores for certain DTSS and DTIS domain and item scores compared with participants without current DT (all *P* < 0.05; Figs. 5a-c and 6a-c).

**Fig. 7:**
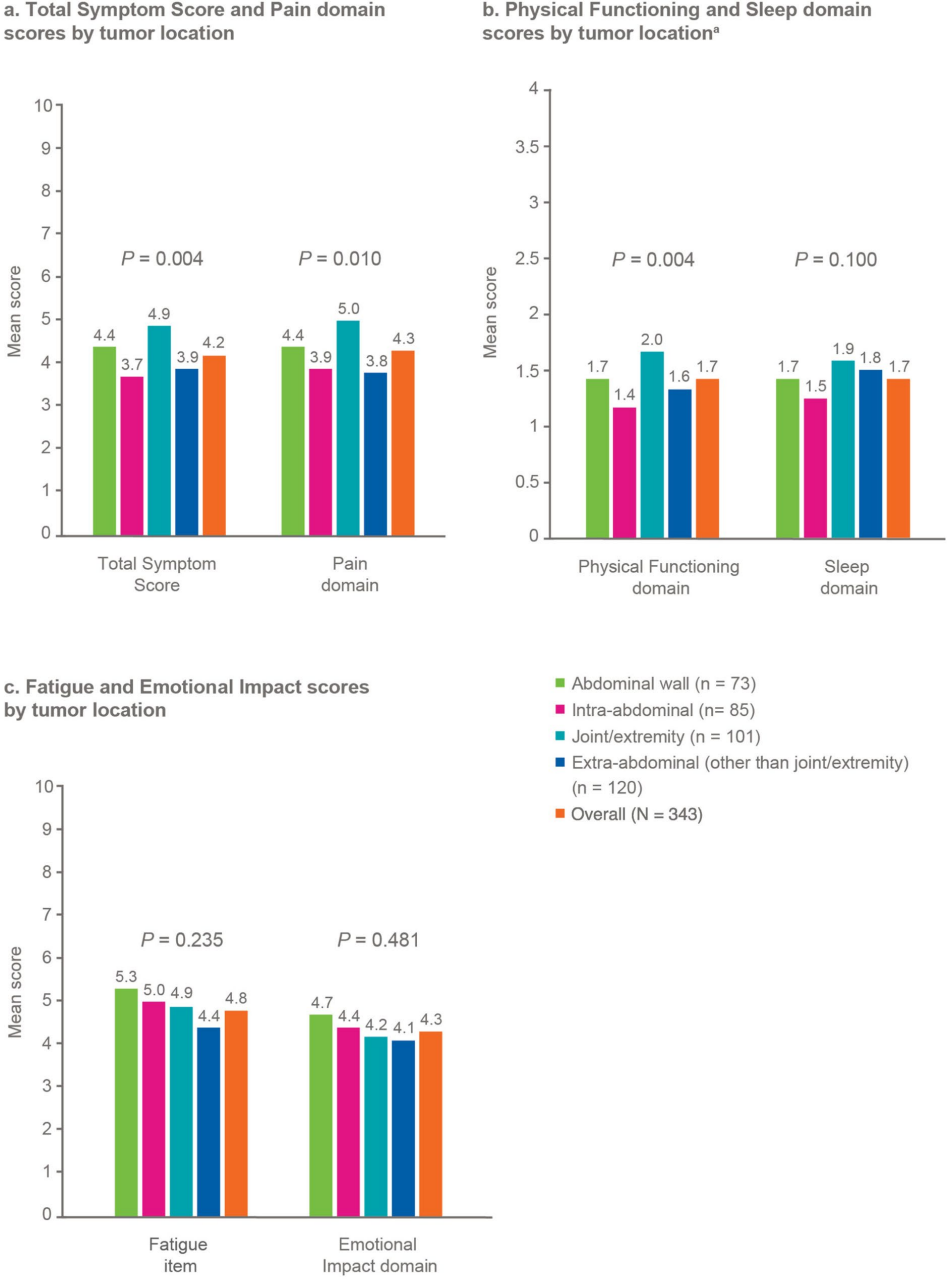
Symptom and impact scores by tumor location. ^a^For the purposes of visibility (not comparison), Fig. 7b is sized in a manner consistent with Fig. 7a and 7c

The symptoms and impact of DT in a joint/extremity were reported to be significantly worse for the DTSS Total Symptom Score, DTSS Pain domain, and the DTIS Physical Functioning domain compared with other DT locations (all *P* < 0.05; Fig. 7a-c).

Conversely, regardless of the current presence of DT, participants reported similar mean scores (*P* > 0.05) for the DTIS Emotional Impact domain (4.4 with current DT vs. 3.9 without current DT) and all Emotional Impact domain item scores, including Fear of Desmoid Tumor Growth/Recurrence (5.8 vs. 5.2), Anger (3.4 vs. 3.1), Anxiety (4.5 vs. 4.0), and Frustration (4.8 vs. 4.0), except Hopelessness (3.7 vs. 2.7; *P* = 0.007; data not shown).

## Discussion

To our knowledge, the DTRF NHS participant population reflects the largest DT survey available and growing international representation of patient-reported DT cases [8]. Demographic data are consistent with a published analysis of four Danish health registries (2009–2018), which reported a median age of 38 years, a predominance of female cases (76%), and extra-abdominal locations as the most common sites for DT (49%; followed by abdominal wall [40%] and intra-abdominal or retroperitoneal regions [8%]) [10].

Individuals with DT often experience a long and uncertain diagnostic journey because of the variable nature of symptom presentation and disease progression [6, 11]. Many clinicians initially fail to recognize the significance of early signs and symptoms, and misdiagnosis is common [3]. Consistent with published data [3, 6, 11], participants in this study commonly (41%) reported receiving an initial misdiagnosis regardless of age, tumor size, and tumor location (factors associated with shorter progression-free survival) [12], and almost half of the participants who reported a misdiagnosis underwent surgery before having a pathology-confirmed DT diagnosis. Differential diagnoses included sarcoma (GIST), benign lesions (e.g., muscle injury, lipoma, colon polyps, postoperative scars, early-onset arthritis, hernia, and Baker’s cyst), and malignant conditions (e.g., lymphoma, vascular tumor, and breast cancer), similar to previously reported data [12–15]. Participants in the current analysis reported an average time of 4 years from symptom onset to definitive DT diagnosis, with significantly longer average times to diagnosis if they were ≤ 30 years old at first symptom or had intra-abdominal DT. Therefore, greater awareness is needed among clinicians to recognize the resemblance of DT to other common tumors, enable prompt diagnosis for young adults, and provide timely DT management.

As shown in this analysis, patients present to their healthcare providers with a wide array of common initial symptoms [7, 12], including unexplained bump, pain, and fatigue. Those with the current presence of DT at the time of survey completion reported significantly worse pain (dull pain, shooting pain), swelling around the tumor, and difficulty moving body areas near the tumor (compared with those without the current presence of DT). Additionally, participants with DT in joints and extremities reported significantly worse pain domain score than participants with tumors at other locations. Pain can further debilitate patients with DT and lead to functional limitations, while the management of pain with narcotics can lead to potential dependency on painkillers [6].

Participants reported receiving multiple treatments for DT, demonstrating the variable nature of the disease [6, 10], an evolving standard of care [16], and the need for individualized treatment [4] based on the patient’s unique clinical situation and preference [14]. Clinicians and patients can find post-DT diagnosis treatment-related decision-making challenging [6], as such, the NCCN Clinical Practice Guidelines in Oncology (NCCN Guidelines^®^) for Soft Tissue Sarcomas and the Desmoid Tumor Working Group (DTWG) Global Consensus Paper Guidelines state that diagnosis and treatment be informed and supported by a multidisciplinary team [14, 16].

More than one-third of participants reported having undergone active surveillance before treatment initiation. Historically, the prognosis of DT has potentially been dependent on multiple patient- and tumor-related factors that include the *CTNNB1* gene mutation. The current data show that participants with *CTNNB1* mutation had similar frequency of medical monitoring compared with participants with FAP. The DTWG recommends performing mutational analysis of DT biopsy specimens to confirm the diagnosis and guide further work-up when appropriate [16].

Surgery was the most common first-line treatment with high postoperative tumor growth or recurrence rates, regardless of tumor location. However, while surgery has historically represented the primary treatment modality for patients with DT, the NCCN Guidelines^®^ and DTWG guidelines no longer support surgery as the first-line option in most situations, given high postsurgical recurrence rates [11, 14, 16, 17], the potential for disfigurement and impaired function [17], and the risk of triggering further aggressive tumor behavior [11]. However, consensus guidance also positions surgery as a primary treatment consideration for patients experiencing complications from sporadic mesenteric DT or for those with non-mesenteric DT that progress despite active surveillance or medical treatment [16].

In this study, participants reported use of a variety of systemic treatments since the initial DT diagnosis, including single or multiple therapies, without specifying sequential or concomitant use. NSAIDs were commonly reported as prior treatment; tyrosine kinase inhibitors (particularly sorafenib) and the chemotherapy agents (methotrexate, vinblastine, doxorubicin, and liposomal doxorubicin) were reported in one-third of participants. The use of NSAIDs and hormone antagonists reported in this study is only recommended for certain circumstances by evidence-based guidelines [14, 16]. After this analysis ended in August 2023 and the United States Food and Drug Administration (FDA) approval of nirogacestat, the NCCN Guidelines and DTWG guidelines were updated to include sorafenib and nirogacestat as Category 1, Preferred systemic regimens [14, 16, 18]. Nirogacestat is an oral, small-molecule, targeted gamma-secretase inhibitor and the only FDA-approved therapy for DT in the United States for the treatment of adult patients with progressing DT who require systemic treatment [18]. Additionally, the NCCN Guidelines were updated to include methotrexate with vinorelbine; methotrexate with vinblastine, imatinib, and liposomal doxorubicin; doxorubicin with or without dacarbazine; and pazopanib as Category 2A regimens [14].

Data collected in this analysis show the broad impact of DT on worsening pain, symptoms, and functioning, which are similar to prior studies, including Gounder et al. (2023) [19] and Husson et al. (2019) [6]. QoL endpoints are important outcome measures for patients with DT [9], especially as these tumors exert a minimal impact on mortality [5] and a significant impact on QoL [6]. As such, treatment goals should include improvements in pain, disease-specific symptom burden, physical functioning, role functioning, psychosocial impacts, and overall health-related QoL [19, 20].

There are some limitations to the study due to the retrospective survey-based approach. The data are based on patient-reported outcomes or those reported by legally authorized representatives. This study shows most participants were White or resided in North America. As a result, data may not be generalizable to patients of other races or geographic locations. Nonetheless, the DTRF NHS represents an ongoing, international research endeavor and, as such, additional data can be expected to provide representation across an expanding range of races and geographic locations. To facilitate further expansion, there are plans to translate the DTRF NHS surveys into Spanish and French.

## Conclusion

The DTRF NHS represents the largest ongoing, international, survey-based study of patients with DT [8]. This analysis builds on data from clinical studies by reporting that participants with DT experience a substantial burden at each stage of the disease. Misdiagnosis is common and can prolong the time to treatment initiation or result in unnecessary surgical resection and treatment. With all treatments examined in this analysis, large proportions of participants still have a current DT and experience postsurgical DT recurrence. Pain, fatigue, sleep changes, and impaired physical functioning all contribute to an impaired QoL. This study highlights the real-world unmet needs of patients and the importance of timely diagnosis and patient-centric treatment strategies and goals – in particular, improvements in pain, disease-specific symptom burden, physical functioning, role functioning, psychosocial impacts, and overall QoL.

## Data Availability

The datasets generated and/or analyzed during the current study are not publicly available and represent proprietary data of the Desmoid Tumor Research Foundation and SpringWorks Therapeutics, Inc.
